# Community knowledge of dementia: a cross-sectional study in the Southeast region of Brazil

**DOI:** 10.1590/1980-5764-DN-2025-0323

**Published:** 2026-01-09

**Authors:** Laura Costa de Oliveira Lima, Bruna de Bonifácio Heck, Nami Dezotti, Julia Mikami Kato, Thiago Issa, Maria Izaura Sedoguti Scudeler Agnollitto, Nereida Kilza da Costa Lima, Julio Cesar Moriguti

**Affiliations:** 1 Universidade de São Paulo, Faculdade Medicina de Ribeirão Preto, Ribeirão Preto SP, Brazil.; 2 Pontifícia Universidade Católica de Campinas, Faculdade de Medicina, Campinas SP, Brazil.; 3 Universidade de Ribeirão Preto, Faculdade de Medicina, Ribeirão Preto SP, Brazil.; 4 Universidade de São Paulo, Faculdade de Economia, Administração e Contabilidade de Ribeirão Preto, Ribeirão Preto SP, Brazil.; 5 Faculdade de Ciências da Saúde de Barretos Dr. Paulo Prata, Barretos SP, Brazil.; 6 Universidade de São Paulo, Faculdade Medicina de Ribeirão Preto, Departamento de Clínica Médica, Ribeirão Preto SP, Brazil.

**Keywords:** dementia, aging, attitude to health, health literacy, cognitive dysfunction, socioeconomic factors, demência, envelhecimento, atitude frente a saúde, letramento em saúde, disfunção cognitiva, fatores socioeconômicos

## Abstract

There is a gap in the literature regarding popular knowledge about dementia. Although dementia affects older individuals more frequently, it does not belong to senescence. Lack of awareness contributes to the normalization of cognitive decline, delaying the search for healthcare services, diagnosis, and treatments.

**Objective:**

Assess the population’s knowledge about dementia and its relationship with socioeconomic characteristics.

**Methods:**

Cross-sectional study with a semi-structured questionnaire administered in person to a convenience sample of residents of Ribeirão Preto, São Paulo, Brazil. Descriptive and inferential statistical analyses were conducted. Significance level: p<0.05.

**Results:**

Three hundred residents were interviewed. Among them, 189 (63%) were unaware of the meaning of dementia. In a multivariable regression, higher income increased the odds of knowing what dementia is compared to the lowest income group (adjusted Odds Ratio [aOR]=4.29, 95% confidence interval [95%CI] 1.40–14.11, *p*=0.013). Working in healthcare (aOR=3.30, 95%CI 1.24–9.66, *p*=0.021) or having relatives in this sector (aOR=2.16, 95%CI 1.16–4.07, *p*=0.016) increased the probability of awareness. Limited awareness of risk factors was observed, along with the normalization of dementia signs and symptoms, which were commonly perceived as part of normal aging.

**Conclusion:**

Significant lack of knowledge on the subject was observed, particularly among socioeconomic vulnerable individuals. Dementia was not widely recognized as a pathological and preventable condition. Given an aging society, this study provides a critical foundation for future public health initiatives and research aimed at improving dementia literacy and promoting early intervention.

## RESUMO.

Há uma lacuna na literatura quanto ao conhecimento da população sobre a demência. Embora afete idosos mais frequentemente, não é inerente ao envelhecimento. A falta de conscientização contribui para a normalização do declínio cognitivo, atrasando a busca por serviços de saúde, o diagnóstico e o tratamento.

**Objetivo:**

Avaliar o conhecimento da população sobre demência e sua relação com características socioeconômicas.

**Métodos:**

Estudo transversal com questionário semiestruturado aplicado presencialmente a uma amostra por conveniência de residentes de Ribeirão Preto, São Paulo, Brasil. Realizaram-se análises estatísticas descritivas e inferenciais. Nível de significância adotado: p<0,05.

**Resultados:**

Trezentos residentes foram entrevistados. Dentre eles, 189 (63%) desconheciam o significado de demência. Na regressão multivariada, maior renda esteve associada a maiores chances de conhecer sobre demência, em comparação ao grupo de menor renda (*Odds Ratio* ajustado [ORa]=4,29; intervalo de confiança de 95% [IC95%] 1,40–14,11; p=0,013]. Atuar na área da saúde (ORa=3,30; IC95% 1,24–9,66; p=0,021) ou ter familiares dessa área (ORa=2,16; IC95% 1,16–4,07; p=0,016) também aumentou a probabilidade de conhecimento. Observou-se conhecimento limitado sobre fatores de risco e uma normalização de sinais e sintomas da demência, frequentemente percebidos como parte da senescência.

**Conclusão:**

Observou-se desconhecimento significativo sobre o tema, especialmente entre indivíduos socioeconomicamente vulneráveis. A demência não foi amplamente reconhecida como condição patológica e prevenível. Diante do envelhecimento populacional, este estudo fornece bases para futuras ações e pesquisas em saúde pública, objetivando ampliar o conhecimento sobre demência e promover intervenções precoces.

## INTRODUCTION

 Dementia is a syndrome characterized by cognitive decline, predominantly progressive, leading to significant impairments in the ability to perform activities of daily living^1,2^. Numerous risk factors for dementia have been identified, with advanced age being the most significant^1^. The increase in population ageing is noteworthy in the scenario of demographic transition in which Brazil and other developing countries find themselves^3^. This trend is expected to result in a substantial rise in the number of dementia cases over the next three to five decades^4^. These statistics are concerning, as dementia is one of the leading causes of disability and dependence among older adults, also affecting families and caregivers^1,5^. 

 Despite the multisectoral relevance of this issue—encompassing public health, social expenditures, and human dignity—, there remains a gap in the literature regarding the level of knowledge about dementia in low and middle-income countries^4,6,7^. Paradoxically, the majority of individuals with dementia reside in these regions^1^. This scenario is particularly worrying, as the development and implementation of effective public awareness campaigns require a clear understanding of the population’s knowledge level and the collective perception of the condition^5^. 

 It is known that, in addition to widespread misinformation about the concept of dementia, there is a misconception that its primary signs and symptoms are inherent to aging and, therefore, natural aspects of senescence^4,5,7-10^. As a result, the early stages of dementia tend to be normalized not only by family members but also by older individuals themselves. The belief that nothing can be done is also prevalent^8,9^. Consequently, diagnosis and referral to appropriate services and treatments are often delayed. Although there is no cure for most cases, much can be done, ranging from the use of pharmacological and non-pharmacological therapies to slow disease progression to initiatives that enhance the life quality of patients, families and caregivers^1^. Therefore, early diagnosis is essential, and its delay due to collective misinformation is unacceptable. In this context, the present study aimed to provide updated data on the knowledge that residents of one of the most populous cities in the state of São Paulo have about dementia. 

## METHODS

### Study design and ethics

 This was a cross-sectional study conducted in Ribeirão Preto, São Paulo, Brazil. The study was approved by the Research Ethics Committee of the Clinical Hospital of the Ribeirão Preto Medical School, Universidade de São Paulo (protocol number 6,044,738; registration number 67854123,1,0000,5440). 

### Data collection

 Data were collected in person from May 2023 to July 2024, through a convenience sample of individuals approached in public spaces, across three socioeconomically distinct areas of Ribeirão Preto: North Zone, South Zone, and Downtown—aiming to ensure a broader representation and reduce sampling bias related to socioeconomic status. 

 All participants read and signed two copies of the informed consent form, retaining one of them. Researchers involved in data collection underwent prior training. The interviews were not recorded and took 10 minutes on average. The researchers verbally asked the questions from a physical printed version of the questionnaire and transcribed the answers by hand exactly as they were stated. Subsequently, data were organized into Excel spreadsheets. 

### Subjects

 Adult residents of Ribeirão Preto, of both sexes, aged 18 years or older, were eligible for inclusion. Exclusion criteria were:

- severe speech impairment preventing oral responses;
- cognitive disorders impairing comprehension of the questions;
- severe hearing impairments;
- visual impairments and illiteracy (absolute or functional) without the presence of a witness.

### Instrument

 The research instrument consisted of a semi-structured questionnaire, developed by the authors specifically for this study, aimed at outlining the socioeconomic profile of the participants and assessing their knowledge of dementia. The latter included questions regarding the concept of dementia, perceptions of key signs and symptoms as either normal or abnormal in aging, as well as knowledge of risk factors and preventive measures. Additionally, the questionnaire included questions about the participants’ social circle, evaluating the presence of dementia cases and individuals working in the healthcare field. There were two open-ended questions: the definition of dementia and examples of preventive measures. All the other 13 questions were close-ended. 

### Data analysis

 A descriptive analysis of the data was performed using Jeffrey’s Amazing Statistics Program—JASP^11^ statistical software. Categorical variables were evaluated using the chi-square test. Age, being the only continuous and parametric variable, was compared between two groups (female and male) using Student’s t-test. 

 Univariate binomial logistic regressions were conducted in the statistical software R (version 4.3.2)^12^, using the packages "readxl", "dplyr" and "broom"—to assess the association between eight independent variables and the likelihood of knowing or not knowing the meaning of dementia (dependent variable). The independent variables analyzed were:

- sex;
- age;
- education level;
- monthly household income;
- the participant’s professional field;
- the presence or absence of close family members or friends working in the healthcare field;
- the presence or absence of close family members or friends with a medical diagnosis of dementia; and
- the city subsector where the interview was conducted.

A multivariable binomial logistic regression was also performed in R using the packages "readxl" and "car" and including seven independent variables to estimate their adjusted effects. The selection of variables included in the model was guided not only by univariate analysis results but also by theoretical relevance. Although some variables did not demonstrate statistical significance in the univariate analysis, they were retained in the final model due to their potential role as confounding factors or effect modifiers in the association between sociodemographic characteristics and dementia knowledge, allowing a more accurate estimation of the independent effects of other variables. 

 Type II Wald χ^2^ tests were used to assess global significance via variance analysis (ANOVA). The significance level was set at p<0.05 for all analyses. 

 To develop a multivariable regression model with statistical power for a sample size of 300 individuals, we used the method proposed by Riley et al.^13^ to calculate the upper limit of parameters. This analysis was conducted with R software using the "psampsize" package, assuming a shrinkage factor of 0.9 and a Nagelkerke R² of 0.15. 

 The model included 12 parameters, complying with the allowable maximum of 18 parameters based on sample size, remaining within the recommended threshold to avoid overfitting. 

 Regarding the binomial classification of dementia knowledge, participants were classified as: "Knows what dementia is" if they provided partially or fully correct definitions; or "Does not know what dementia is" if they had never heard of the term, were unable to define it, or gave incorrect responses. Responses were independently assessed by two authors, with discrepancies resolved by a third. 

## RESULTS

 Data were collected in person from 300 individuals approached in three subsectors of Ribeirão Preto (SP): Central (n=100), Northern Zone (n=100), and Southern Zone (n=100). The distribution of data obtained from the questionnaires is in Table 1. The mean age of the total sample was 44.8±17.6 years. There was no significant difference in age between males and females (43.8±17.8 vs. 45.78±17.3 years, p=0.332). Among all participants, 111 individuals (37%) had knowledge about the subject. The remaining 189 (63%) respondents did not know the meaning of dementia. Regarding the definitions of dementia provided by the residents, the most frequently mentioned were: memory loss/forgetfulness, loss of cognitive ability, mental confusion, loss of brain capacity, loss of reasoning, and "being lost." 

**Table 1:** Distribution of data obtained from the questionnaire—n (%).

Question			n (%)
Sex	Male		149 (49.7)
	Female		151 (50.3)
Age (years old)	18–39		121 (40.3)
	40–59		109 (36.3)
	≥60		70 (23.3)
*Monthly household income	1–3		161 (53.7)
	3–9		99 (33)
	>9		33 (11)
	Did not want to disclose		7 (2.3)
Education level	Incomplete elementary/no formal education		44 (14.7)
	Complete elementary		46 (15.3)
	Complete high school		131 (43.7)
	Complete higher education		79 (26.4)
Labor	Healthcare field		30 (10)
	Other fields		270 (90)
Close relative/friend working in healthcare	Yes		102 (34)
	No		198 (66)
Dementia syndrome signs and symptoms considered as natural/expected consequences of aging	Spatio-temporal disorientation		137 (45.7)
	Speech difficulties		161 (53.7)
	Behavioral and mood changes		206 (68.7)
	Misplacing objects		206 (68.7)
	Memory loss		208 (69.3)
	Loss of ability to work and/or engage in social activities		215 (71.7)
	Loss of ability to perform IADLs		241 (80.3)
+Opinion regarding the existence of dementia prevention measures	No		74 (24.7)
	Yes, but could not provide examples		45 (15)
	Yes, could provide examples		181 (60.3)
Would seek medical assistance	Yes		242 (80.7)
	No		58 (19.3)
Has ever heard of the term "dementia"	No		61 (20.3)
	Yes		239 (79.7)
Knows the meaning?	Answered no		81 (33.9)
	+Answered yes		158 (66.1)
		Inadequate answer	47 (19.6)
		Adequate answer	25 (10.5)
		Partially adequate answer	86 (35.9)
§Close relative/friend with a medical diagnosis of dementia	Yes		58 (24.3)
	No		181 (75.7)
||Dementia can occur	At any age		51 (32.3)
	At any age, but primarily in older adults		75 (47.5)
	Only in older adults		32 (20.2)
§Frequency with which each factor was considered a risk for dementia	Hearing impairment		22 (13.7)
	High cholesterol		27 (17.3)
	High blood pressure		31 (19.6)
	Limited access to education		36 (22.5)
	Diabetes		42 (26.5)
	Obesity		43 (27.5)
	Head injuries		52 (33)
	Low social interaction		53 (33.3)
	Smoking		62 (39.3)
	Physical inactivity		67 (42.5)
	Excessive alcohol consumption		70 (44.2)
	Depression		74 (47.1)

Abbreviations: IADLs, Instrumental Activities of Daily Living.

Notes: *in minimum wages; +open-ended question; §this question was asked to 239 participants; it was not asked to the 61 participants who had never heard of the term dementia; ||question only asked to those who said that knew what dementia was (158 participants).

### Univariate analysis

 In the univariate models (Table 2), several variables demonstrated statistically significant associations with knowledge about dementia. Individuals with a monthly income between three and nine minimum wages (MW) had higher odds of knowing about dementia (odds ratio [OR]=4.84, 95% confidence interval [CI] 2.80–8.50, *p*<0.001) compared to those earning 1–3 MW. The association was even stronger for individuals with income greater than nine MW (OR=9.27, 95%CI 4.12–22.27, *p*<0.001). 

**Table 2:** Odds ratios and 95% confidence intervals from univariate logistic regression models.

Variable*		OR	95%CI	Std. Error	z-value	p-value
Monthly income§	(Intercept)	0.248	0.166–0.360	0.198	-7.059	<0.001
	3–9	4.838	2.802–8.496	0.282	5.582	<0.001
	>9	9.272	4.118–22.266	0.427	5.213	<0.001
City subsector the interview was conducted	(Intercept)	0.282	0.176–0.453	0.241	-5.24	<0.001
	South	3.273	1.770–6.051	0.314	3.78	<0.001
	Downtown	2.567	1.384–4.762	0.315	2.99	0.003
Age	(Intercept)	0.571	0.392–0.823	0.189	-2.961	0.003
	40–59 yo	1.097	0.642–1.875	0.273	0.339	0.734
	≥60 yo	1.034	0.559–1.899	0.311	0.108	0.914
Professional field	(Intercept)	0.509	0.393–0.654	0.130	-5.193	<0.001
	Healthcare field	6.461	2.799–16.815	0.451	4.138	<0.001
Family/friends working in Healthcare	(Intercept)	0.385	0.280–0.522	0.159	-6.022	<0.001
	Yes	3.293	2.006–5.456	0.255	4.677	<0.001
Family/friends with dementia	(Intercept)	0.664	0.488–0.897	0.155	-2.642	0.008
	Yes	4.321	2.273–8.600	0.338	4.334	<0.001
Sex	(Intercept)	0.678	0.488–0.936	0.166	-2.345	0.019
	Male	0.768	0.479–1.227	0.239	-1.104	0.270
Education level	(Intercept)	0.273	0.123–0.546	0.376	-3.455	<0.001
	Complete Elementary	0.772	0.262–2.242	0.541	-0.478	0.632
	Complete High School	1.919	0.873–4.580	0.419	1.556	0.120
	Complete Higher Education	6.322	2.746–15.785	0.443	4.166	<0.001

Abbreviations: OR, odds ratio; CI, Confidence Interval; yo, years old.

Notes: *The categories of each independent variable are being compared to the reference category. The reference categories are: (1) Monthly income: 1–3 minimum wages; (2) City subsector: north; (3) Age: 18–39 years old; (4) Professional field: other fields (not healthcare); (5) Family/friends working in Healthcare field: no; (6) Family/friends with dementia: no; (7) Sex: female; (8) Education level: incomplete elementary/no formal education. § in minimum wages

 The residents of the Downtown (OR=2.57; 95%CI 1.38–4.76; p=0.003) and South zones (OR=3.27; 95%CI 1.77–6.05; p<0.001), both wealthier subsectors of the city, had higher odds of knowing about the topic when compared to the residents of the North zone. Educational attainment also showed a gradient effect: participants with complete higher education were more likely to report knowing about dementia (OR=6.32, 95%CI 2.75–15.79, p<0.001) compared to those with incomplete or no formal education. 

 Participants working in the healthcare field had significantly increased odds of knowledge (OR=6.46, 95%CI 2.80–16.82, p<0.001), as did those who reported having family members or friends working in healthcare (OR=3.29, 95%CI 2.01–5.46, p<0.001). Furthermore, having relatives or close friends with dementia was strongly associated with greater odds of knowledge (OR=4.32, 95%CI 2.27–8.60, p<0.001). Age and sex were not significantly associated in the univariate models. 

### Multivariable analysis

 As shown in Table 3, Type II deviance tests confirmed that the global adjusted effect of income (p=0.017), professional field (p=0.016), having relatives/friends in healthcare (p=0.015) and having known cases of dementia among close contacts (p<0.001) were significant contributors to the model. 

**Table 3:** Results of multivariable binomial logistic regression: deviance analysis and multicollinearity assessment.

Predictor Variable	LR Chisq	Df	p-value	GVIF	GVIF^(1/(2×Df))
Income	8.178	2	0.017	1.703	1.142
Education level	0.995	3	0.803	1.803	1.103
Professional field	5.817	1	0.016	1.159	1.077
Relatives/friends in Healthcare	5.879	1	0.015	1.057	1.028
Dementia cases in family/friends	15.985	1	<0.001	1.081	1.040
Age	0.649	2	0.723	1.252	1.058
Sex	1.124	1	0.289	1.124	1.060

Abbreviations: LR Chisq, Likelihood Ratio Chi-square; Df, degrees of freedom; GVIF, Generalized Variance Inflation Factor; GVIF^(1/(2×Df)): scaled GVIF.

 As seen in Table 4, several of the associations remained significant in comparison to univariate models, although effect sizes were attenuated. Income continued to be a significant predictor: individuals earning 3–9 MW (aOR=2.46, 95%CI 1.19–5.16, p=0.016) and those earning >9 MW (aOR=4.29, 95%CI 1.40–14.11, p=0.013) had increased odds of knowing what dementia is compared to the lowest income group. 

**Table 4:** Multivariable binomial logistic regression adjusted odds ratios.

		aOR	95% CI	p-value
*Variable	(Intercept)	0.409	0.122–1.269	0.131
Monthly income	3–9	2.456	1.185–5.163	0.016
	>9	4.292	1.397–14.105	0.013
Education level	Complete Elementary	0.596	0.154–2.281	0.448
	Complete High school	0.718	0.232–2.292	0.567
	Complete Higher education	0.931	0.260–3.356	0.912
Professional field	Healthcare field	3.300	1.243–9.663	0.021
Friends/family working in Healthcare	Yes	2.159	1.159–4.069	0.016
Family/friends with dementia	Yes	4.258	2.067–9.165	<0.001
Age	40–59 yo	0.735	0.342–1.551	0.423
	≥60 yo	0.842	0.368–1.916	0.682
Sex	Male	0.710	0.373–1.337	0.290

Abbreviations: aOR, adjusted odds ratio; yo, years old; CI, confidence interval.

Notes: *The categories of each independent variable are being compared to the reference category. The reference categories are: (1) Monthly income: 1–3 minimum wages; (2) Education level: incomplete elementary/no formal education; (3) Professional field: other fields (not healthcare); (4) Family/friends working in Healthcare field: no; (5) Family/friends with dementia: no; (6) Age: 18–39 years old; (7) Sex: female.

 The effect of working in the healthcare field remained significant (aOR=3.30, 95%CI 1.24–9.66, p=0.021), as did having family or friends in the healthcare sector (aOR=2.16, 95%CI 1.16–4.07, p=0.016). Notably, having relatives or friends with dementia was a strong independent predictor (aOR=4.26, 95%CI 2.07–9.17, p<0.001), suggesting that personal exposure may importantly influence awareness. 

 In contrast, education level, age, and sex were not independently associated with dementia knowledge in the multivariable model. This suggests that their apparent effects in the univariate models may have been confounded or mediated by other variables such as income or healthcare exposure. 

 Generalized variance inflation factors (GVIFs) and their scaled versions (GVIF^1/(2×Df)) indicated low multicollinearity among predictors (Table 3). All scaled GVIF values were close to 1 (range: 1.028–1.142), well below the commonly used thresholds of concern (e.g., >2), suggesting that multicollinearity did not adversely affect model estimates. 

### Signs and symptoms of dementia syndrome

 It was observed that a significant portion of respondents considered the main signs and symptoms of dementia syndrome part of physiological aging (senescence) rather than pathological aging (senility) (Table 1). 

 Only ten participants (3.3%) did not consider any sign or symptom of dementia as a natural consequence of aging. Half of them were healthcare professionals, four had relatives or close friends working in the healthcare field, and three had family members with a medical diagnosis of dementia. 

### Seeking medical assistance

 One of the items in the questionnaire asked whether the respondent would seek medical assistance if they or a relative/friend experienced any dementia signs or symptoms. 

 There was no difference in seeking medical assistance across different age groups (p=0.71). However, there was a significant sex difference (female=88.1% vs. male=73.2%; p<0.001). In addition, individuals from higher-income households reported a higher rate of affirmative responses regarding seeking medical assistance, compared to those with lower income (1 to 3 MW=73.9%; 3 to 9 MW=87.9%; 9 to 15 MW: 100%; +15 MW=83.3%; p=0.004). 

### Risk factors for dementia

 A significant lack of knowledge about risk factors for dementia was observed. Only six participants identified all factors as being associated with dementia risk. Among them, half were male, and four worked in the healthcare sector. Additionally, four had relatives or close friends working in healthcare, and two had family members or close friends with a medical diagnosis of dementia. 

### Prevention and reduction of signs and symptoms

 The frequency of each different opinion regarding dementia prevention measures can be seen in Table 1. The most frequently mentioned preventive measures included physical activity, intellectual activities (such as reading and games), a healthy and balanced diet, active social interaction, as well as family and social support for older adults. The less frequently mentioned measures included regular medical check-ups, medication, the use of eyeglasses, spiritual well-being, neurological treatment, vitamin intake, geriatric follow-ups, psychological and psychiatric support, lifelong learning, quality sleep, and avoiding smoking and alcohol consumption. 

## DISCUSSION

 This study was among the first to assess dementia-related knowledge in Brazil through in-person data collection, with a deliberate effort to include participants from all income and education levels, while conducting it across three socioeconomically distinct sectors of the city. 

 Analysis of the results revealed that more than half of the surveyed population lacks knowledge about dementia, particularly those with lower income and educational levels. 

 These points situate Brazil within global trends and support the external validity of our findings without overstating cross-country comparability. 

 Even among the 111 respondents (37%) classified as knowledgeable about the topic, a significant proportion had a fragmented and simplified understanding of the subject. The concepts of dementia presented by respondents were incomplete and, in most cases, based on personal experiences or common perceptions. The fact that women seek medical assistance more often than men reflects a well-documented trend that female sex individuals generally have greater health concerns and make more use of healthcare services^14,15^. 

 Most individuals who were aware of dementia, despite recognizing its components (such as memory loss, confusion, and cognitive decline), lacked a comprehensive and structured understanding of dementia as a syndrome distinct from normal aging (senescence) and subjectable to preventive measures. Modifiable risk factor control has the potential to prevent up to one-third of dementia cases^6^, which makes the general lack of awareness regarding these factors even more concerning. 

 A recent study conducted using the public database of the Brazilian Longitudinal Study of Aging (ELSI-Brazil) found that the total weighted population attributable fraction (PAF) for ten risk factors evaluated in the country was 50.5%. The highest PAFs were hearing loss (14.2%), physical inactivity (11.2%), hypertension (10.4%) and low education level (9.5%)^16^. As shown in Table 1, all factors with the highest PAFs were poorly recognized by the population in the present study as risk factors for dementia. Hearing impairment (13.7%), hypertension (19.6%), and physical inactivity (42.5%) were among the least recognized. 

 The results of both univariate and multivariable logistic regression analyses indicated that working in the healthcare field, having family or friends employed in the healthcare sector, and having family or friends diagnosed with dementia significantly increased the likelihood of knowing what dementia is. These findings are expected, as individuals with such backgrounds are likely to be more frequently exposed to health-related information. However, these results should not diminish concerns regarding the overall level of knowledge, even within the healthcare sector. National literature reveals troubling levels of misinformation among healthcare professionals. A 2024 study reported that 33.3% of healthcare professionals perceive dementia as part of the aging process^7^. Another study conducted with a sample of primary care physicians in the Southern region of Brazil obtained less than 50% of correct answers in a knowledge quiz^17^. These findings highlight the urgent need for targeted educational interventions, even among those presumed to be better informed. 

 Furthermore, data reveals disparity in dementia awareness among residents of Ribeirão Preto, highlighting the impact of socioeconomic variables on the dissemination and understanding of health information. There is a strong correlation between socioeconomic status and health literacy^18-21^. In wealthier areas, the population generally has more opportunities to acquire medical and preventive health knowledge, whether through more frequent health campaigns, greater internet access, or interactions with healthcare professionals within their social circles^22^. 

 Conversely, individuals facing socioeconomic vulnerability tend to have fewer comprehension of chronic diseases and lower adherence to health promotion and prevention programs^23^. Moreover, due to lower educational attainment, there exists an immeasurable gap between disseminated information and the collective understanding of such content. This creates a cycle of misinformation and lack of awareness, potentially impacting the control of modifiable risk factors, early diagnosis, and long-term care planning. 

 The knowledge trends found in this study were consistent with the global literature^6,24-26^, which generally shows only moderate to low public understanding, as well as poor recognition of prevention measures. There is also a consensus among studies that dementia is widely considered an expected part of normal aging and that higher education is associated with better performance on the topic. It is challenging, though, to make comparisons with the available studies on the topic, since very few were conducted in low- to middle-income countries. 

 A large systematic review^24^ on the topic included 40 studies from 15 countries, only one of which —with 73 participants—was conducted in Brazil^27^. Another substantial systematic review with 33 studies^6^ did not include Brazilian research. In both reviews, most of the studies were conducted in the USA and developed countries of Western Europe. 

 This study has several limitations, such as its sample size and the semi-structured nature of the questionnaire, which leaves room for subjective interpretations of open-ended responses. Additionally, although the data were collected in three different socioeconomic regions of the city of Ribeirão Preto, as the study was conducted solely among residents of a single city and with a convenience sample, the findings cannot be generalized. 

 Given the reality of an aging society, it is imperative to address dementia in a systematic and comprehensive manner, with the support of healthcare professionals and policymakers and an emphasis on vulnerable populations^28^. Although dementia predominantly affects older adults, it is not an inherent part of aging^29^. The collective lack of awareness regarding the topic normalizes debilitating cognitive decline, ultimately delaying the search for healthcare services, diagnosis, and appropriate treatment. The findings of this study underscore the urgent need for targeted educational strategies and provide a critical foundation for guiding future public health initiatives and research aimed at improving dementia literacy, reducing disparities, and promoting early intervention. 

## Data Availability

The datasets generated and/or analyzed during the current study are available from the corresponding author upon reasonable request.
